# Putaminal-cortical circuits predict response of bilateral deep brain stimulation of the subthalamic nucleus in the primary Meige syndrome after 5 years

**DOI:** 10.1093/braincomms/fcaf042

**Published:** 2025-02-08

**Authors:** Ning Wang, Yifeng Wu, Chen Yao, Dawei Meng, Haoran Zhang, Qinxiu Cheng, Xiaodong Zhang, Hailiang Shen, Yingqi Lu, Lin Wang, Jinping Xu

**Affiliations:** Department of Neurosurgery, Aviation General Hospital, Beijing 100012, China; Institute of Biomedical and Health Engineering, Shenzhen Institutes of Advanced Technology, Chinese Academy of Sciences, Shenzhen 518055, China; Southern University of Science and Technology, Shenzhen 518055, China; Department of Neurosurgery, The National Key Clinic Specialty, Shenzhen Key Laboratory of Neurosurgery, The First Affiliated Hospital of Shenzhen University, Shenzhen Second People’s Hospital, Shenzhen 518035, China; Department of Neurosurgery, Aviation General Hospital, Beijing 100012, China; Institute of Biomedical and Health Engineering, Shenzhen Institutes of Advanced Technology, Chinese Academy of Sciences, Shenzhen 518055, China; Southern University of Science and Technology, Shenzhen 518055, China; Institute of Biomedical and Health Engineering, Shenzhen Institutes of Advanced Technology, Chinese Academy of Sciences, Shenzhen 518055, China; Institute of Biomedical and Health Engineering, Shenzhen Institutes of Advanced Technology, Chinese Academy of Sciences, Shenzhen 518055, China; Institute of Biomedical and Health Engineering, Shenzhen Institutes of Advanced Technology, Chinese Academy of Sciences, Shenzhen 518055, China; Institute of Biomedical and Health Engineering, Shenzhen Institutes of Advanced Technology, Chinese Academy of Sciences, Shenzhen 518055, China; Department of Neurosurgery, Aviation General Hospital, Beijing 100012, China; Institute of Biomedical and Health Engineering, Shenzhen Institutes of Advanced Technology, Chinese Academy of Sciences, Shenzhen 518055, China

**Keywords:** deep brain stimulation, subthalamic nucleus, Meige syndrome, structural MRI, voxel-based morphology

## Abstract

The deep brain stimulation (DBS) in the subthalamic nucleus (STN) has attracted more attention for primary Meige syndrome due to easier target location and lower power consumption. However, potential and reliable preoperative predictors of longitudinal outcomes of STN-DBS to guide therapeutic decisions remain largely unexplored. Herein, we used preoperative structural MRI and Burke–Fahn–Marsden Dystonia Rating Scale (BFMDRS) from 55 patients with primary Meige syndrome who finished STN-DBS after 5 years. They were further classified into response (*n* = 23) and super-response (*n* = 32) based on the improvement rates of BFMDRS. Voxel-based morphology, partial correlation analyses, receiver operating characteristic (ROC) analyses and support vector machine were performed. We identified that improved rates of BFMDRS were 63, 71.97, 76.64, 79.51, 81.02, 81.36, 81.16, 80.80 and 80.93% at 1, 3, 6, 12, 18, 24, 36, 48 and 60 months after STN-DBS, respectively, and remained steady across 1–5 years. Further voxel-based morphology analyses revealed significantly lower grey-matter volume in the right hippocampus, left putamen, right supramarginal gyrus and left superior frontal gyrus in response when compared with super-response. The grey-matter volumes in the left putamen, right supramarginal gyrus and left superior frontal gyrus were not only positively correlated with improvement rates of BFMDRS after STN-DBS for 5 years in the primary Meige syndrome, but also presented a reliable classification ability in distinguishing response and super-response (area under curve = 0.855). These results suggested that STN-DBS is an effective treatment for primary Meige syndrome, and preoperative grey-matter volume of putaminal-cortical circuits could be used as potential biomarkers to predict longitudinal outcomes.

## Teaser

Wang *et al*. reported that deep brain stimulation in the subthalamic nucleus (STN) is an effective treatment for primary Meige syndrome with an improved rate of motor scores nearly 80% after 5 years. Moreover, the preoperative grey-matter volumes of putaminal-cortical circuits could be used as potential biomarkers to predict longitudinal outcomes of deep brain stimulation in the STN.

## Graphical Abstract

**graphical abstract:**
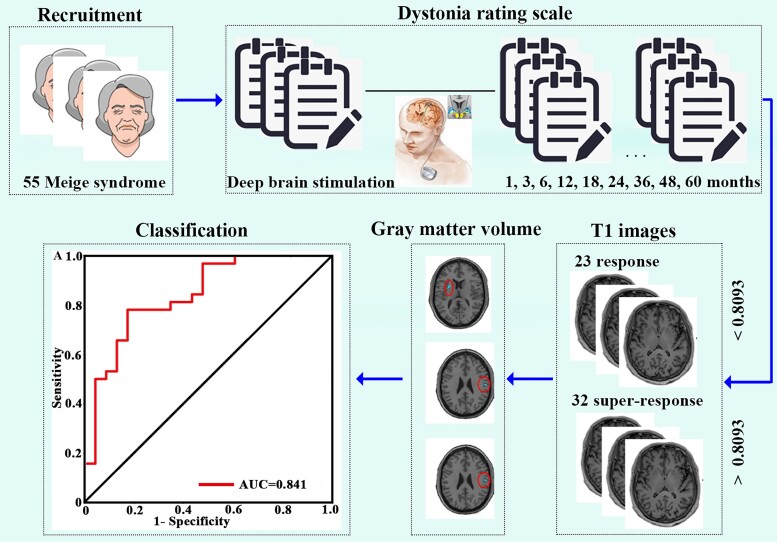

## Introduction

Primary Meige syndrome is a common type of segmental dystonia, which was characterized by a bilateral involuntary contraction of the orbicularis oculi muscles at the early stage, and then spread to perioral and cervical muscles.^1^ As for those medically refractory patients or late-stage patients who have failed initial therapy with botulinum toxin, deep brain stimulation (DBS) has been widely accepted as an effective treatment, for which the globus pallidus internus (GPi) and subthalamic nucleus (STN) were used as surgical options.^2^ Based on previous reviews,^3,4^ there was significant improvement in the Burke–-Fahn–Marsden Dystonia Rating Scale (BFMDRS) postoperatively at the last follow-up visit both in the GPi and STN group, and both groups showed no significant difference in BFMDRS motor improvement thereafter in the Meige syndrome.

Although the GPi-DBS is a common choice for Meige syndrome for a long time, STN-DBS has attracted more attention on the neuromodulation for Meige syndrome in recent years for the following main reasons.^1,5-7^ First, many patients with Meige syndrome especially the older ones showed several infarcts in the basal ganglia, including GPi on the MRI. It has been reported that the normal functions of activating contacts might be affected by these infarcts at this site. Second, it might take several days, weeks or even months to get a positive response after GPi-DBS for some patients, making it more complicated for programming after surgery, whereas the outcomes of STN-DBS are relatively quick and obvious. Finally, using other structures or nuclei such as red nucleus as a reference, the STN is easier to locate in the MRI than GPi, making the location more accuracy. A recent study^8^ evaluated the effects of STN-DBS on motor symptoms in patients with primary Meige syndrome showed a mean improvement of 66.8% at 3 years after neurostimulation using 30 patients, aligning with previous reports showing improvement rates ranging from 56 to 93%.^9-12^ As we noticed, outcomes varied among patients, some responded very well, whereas others might be low responders or even non-responders to STN-DBS. If the neurologists had an accurate prognostic test of response to DBS, more personalized and optimal surgery could be performed to improve clinical outcome and avoid potential side-effects at a specific time.

Although some retrospective studies showed several clinical and demographic factors, such as baseline severity,^3,7^ disease duration,^13^ age at surgery^14^ or stimulation targets,^15^ might serve as predictors for the prognosis of DBS, they were unreliable and insignificant in other studies.^3^ Standard structural MRI was a routine pre-assessment for patients, which was superior to functional and diffusional metrics in disease classification and prediction.^16^ Specific structural features have been reported to serve as potential biomarkers in accumulating prediction tasks.^17-19^ Particularly, one previous study using a large cohort of 82 dystonia patients demonstrated that individual grey-matter atrophy patterns can predict poorer GPi-DBS outcome,^20^ whereas another study demonstrated that connectivity between the primary motor putamen and the posterior GPi limb could predict the GPi-DBS outcomes in cervical dystonia.^21^ In addition, the grey-matter volume (GMV) in the left Lobule VIIIb could predict the outcomes of STN-DBS surgery.^22^ However, this study was performed to focus on the cerebellum, potentially overlooking the useful information of the cerebrum. As we all know, there are a wide range of structural abnormalities in the cerebrum in the Meige syndrome when compared with healthy controls, whether these MRI-based grey-matter metrics can be useful to better predict the outcomes of STN-DBS surgery has not been addressed.

Therefore, in the current study, we used preoperative structural MRI and longitudinal clinical evaluations from 55 patients with primary Meige syndrome who finished STN-DBS after 5 years, and aimed to: (i) evaluate the longitudinal outcomes of STN-DBS in the Meige syndrome after 5 years; (ii) explore the group differences of structural patterns based on whole-brain voxel-based morphology (VBM) between response and super-response and (iii) test whether these structural indexes could be used as biomarkers to predict longitudinal outcomes of STN-DBS in the Meige syndrome after 5 years.

## Materials and methods

### Participants

Fifty-five patients with primary Meige syndrome who underwent bilateral STN-DBS and finished 5 year’s longitudinal evaluations were enrolled in the current study. Based on the diagnostic criteria described by Pakkenberg *et al*.,^23^ all patients were diagnosed by two experienced neurologists (L.W. and N.W.) specialized in movement disorders. The inclusion criteria were: (i) age between 18 and 75 years and (ii) substantial motor impairment. The exclusion criteria were: (i) secondary dystonia; (ii) severe cognitive impairment, dementia or psychiatric disorders; (iii) neurologic disorders (craniocerebral trauma, stroke, brain stem demyelination, encephalitis or cerebral hypoxia) other than dystonia and (iv) contraindications to MRI. This study was approved by the Aviation General Hospital institutional review board, and all patients provided written informed consent.

### MRI image acquisition

The preoperative three-dimensional (3D) T_1_-weighted data were collected using a 3T MRI scanner (Tim Trio; Siemens, Erlangen, Germany) with a magnetization-prepared rapid-acquisition gradient-echo pulse sequence before STN-DBS. The main parameters were repetition time = 2300 ms; echo time = 2.91 ms; inversion time = 900 ms; flip angle = 9°; matrix dimensions = 256 mm × 256 mm and voxel size = 1 × 1 × 1 mm^3^.

### STN-DBS electrode implantation

All patients with Meige syndrome underwent bilateral STN-DBS surgery after careful preoperative examinations. The surgical procedure was the same as our previous studies.^15,24^

**Figure 1:**
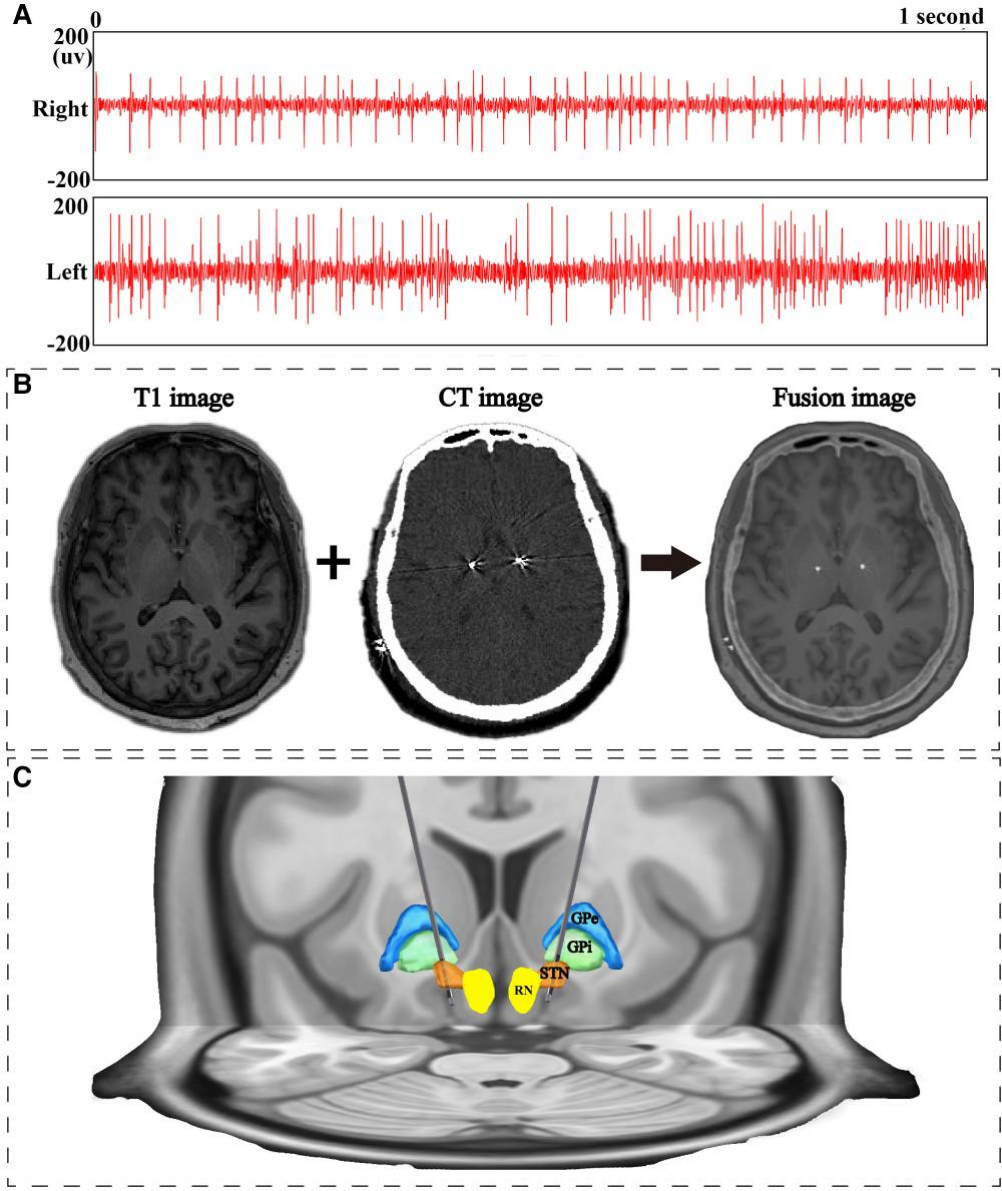
**Lead placement accuracy assessed by microelectrodes and MRI–CT fusion images.** (**A**) The image presents high-amplitude and high-frequency discharges of STN neurons on both sides of a patient (*n* = 1), recorded via microelectrodes. (**B**) The image shows the correct placement of electrodes determined by preoperative T_1_ MRI, postoperative CT and a fusion of both images. (**C**) The image is a 3D reconstruction using Lead-DBS software (https://www.lead-dbs.org/), incorporating preoperative T_1_ MRI and postoperative CT images. GPe, external globus pallidus; GPi, internal globus pallidus; RN, red nucleus; STN, subthalamic nucleus.

On the day of the operation, the patients were secured in a Leksell Micro-Stereotactic System (Elekta Instrument AB, Stockholm, Sweden). The target location for the STN was determined based on anterior–posterior commissure and relative location to the red nucleus in the preoperative MRI-T_1_ images, and adjusted using MRI-T_2_ or SWI if needed, making the electrode go through the dorsal lateral long axis of STN. Bilateral implantation of quadripolar electrodes (electrode L301; PINS Medical Co., Ltd, Beijing, China), which had four contacts (contact height of 1.5 mm, a spacing of 1.5 mm between each contact and a diameter of 1.3 mm), was implanted into the STN by one experience neurosurgeon (L.W.). Microelectrode recordings (NeuroNav, Alpha Omega) were used during surgery to confirm the accuracy of the target. The STN was identified based on the relatively irregular neuronal discharge pattern of high-amplitude and high-frequency activity observed intraoperatively (Fig. 1A). In addition, one CT scan was performed to confirm the accuracy of the target and to exclude the possibility of intracranial haemorrhage.

Then, an implantable pulse generator (IPG; G102, G102R or G102RZ; PINS Medical Co., Ltd) was implanted into the subclavicular region, and the electrodes were then connected to the IPG under general anaesthesia. A postoperative thin-layer CT scan 7–8 days after surgery and preoperative MRI fusion images were used to verify the exact location of the electrode positions (Fig. 1B and C).

The IPG programming was initiated ∼4 weeks after surgery, allowing for the selection of optimal contacts and stimulation parameters based on symptom changes and adverse effects. In our study, the stimulation was initially performed at a frequency of 130 Hz with a pulse width of 60 μs. The voltage was increased from 0 to 1.0 V with an increment of 0.1 V and then slowly increased beyond 1.0 V in one step of 0.05–0.1 V. The optimal parameters were selected when patients achieved satisfactory improvement with minimal side-effects. The patients were only programmed once, and they did not receive refined programming at each follow-up time.

### Clinical outcomes

Baseline assessments, including the patients’ age, sex and disease durations, were obtained from all patients by face-to-face interviews before the surgery. Dystonic symptoms were assessed using the BFMDRS at 1, 3, 6, 12, 18, 24, 36, 48 and 60 months after STN-DBS. BFMDRS improvement rates were calculated as (BFMDRS before STN-DBS − BFMDRS after STN-DBS)/BFMDRS before STN-DBS. These patients were further divided into two groups based on their sustained clinical improvement rate of STN-DBS after 5 years, including response (lower than mean improvement rate of 80.93%) and super-response (higher than mean improvement rate of 80.93%).

### Voxel-based morphology

The T_1_-weighted images of all participants were pre-processed using the Data Processing and Analysis of Brain Imaging toolbox (http://rfmri.org/dpabi). First, the quality of each image was visually checked, and none of the participants was excluded due to poor imaging quality. T_1_ images were processed using the New-segment and DARTEL with the following steps. Specially, all images were segmented into three tissues, including the grey matter, white matter and cerebrospinal fluid, and transformed into a standard Montreal Neurological Institute (MNI) space. Subsequently, these images were modulated to preserve the regional volume information. Finally, the modulated grey-matter images were smoothed with a 6 mm full-width at half maximum for subsequent morphological analyses.

Then, whole-brain voxel-wise GMV between response and super-response was compared using two-sample *t*-tests, with age, sex, estimated total intracranial volume (eTIV), disease durations and BFMDRS before surgery as the covariates to map the overall GMV alterations (voxel level *P* < 0.001, cluster size > 40).

For regions showing GMV alterations, we calculated the mean GMV in these regions for each group, and compared them using two-sample *t*-tests with age, sex, eTIV, disease durations and BFMDRS before surgery as the covariates in SPSS 25.0 (IBM, Armonk, NY, USA). Then, we also examined the relationships between preoperative GMV of significant clusters and BFMDRS improvement rates in the patients with Meige syndrome using partial correlation analyses after adjusting for age, sex, eTIV, disease durations and BFMDRS before surgery. Finally, we also examined the relationships across preoperative GMV of significant clusters to determine whether they were related to each other using Pearson’s correlation. The significance level was set at *P* < 0.05/4 = 0.0125, Bonferroni corrected for multiple comparisons.

### Receiver operating characteristic

The ROC analyses were performed to determine to what extent the GMV of clusters related to BFMDRS improvement rates can distinguish response from super-response using SPSS 25.0 (IBM). The mean GMV of all three clusters [left putamen, right supramarginal gyrus (SMG.R) and left superior frontal gyrus (SFG.L)] was used as input features in the ROC analyses since they are not only significantly decreased in the response when compared with super-response, but also positively correlated with improvement rates of BFMDRS after STN-DBS for 5 years. The classification ability was evaluated by the area under the curve (AUC).

### Support vector machine

Additionally, we employed the support vector machine (SVM) within the LIBSVM (https://www.csie.ntu.edu.tw/~cjlin/libsvm/) using Python 3. The mean GMV of all three clusters (left putamen, SMG.R and SFG.L) were used as input features in the SVM. Given that SVM is highly sensitive to feature scaling, we standardized these three features before modelling. The radial basis function was used as the Kernel function to address non-linear relationships in the model architecture. We used the leave-one-out method, with the leaving one data as testing and the remaining data as training. During training, 10-fold cross-validation was used in grid research to fit the optimal model. Finally, we plotted the AUC to evaluate the performance of SVM model.

### Statistical analyses

Differences in the age, disease durations, eTIV and BFMDRS after STN-DBS for 5 years between two groups were assessed by two-sample *t*-tests. Sex differences between the two groups were compared using the Pearson χ^2^ test. BFMDRS differences between each time point and before STN-DBS were compared using the paired two sample *t*-tests. The relationships between BFMDRS improvement rates and clinical variables (age, sex, eTIV, disease durations and BFMDRS before surgery) were performed using Pearson’s correlation in the patients with Meige syndrome. For all of the analyses, we used SPSS 25.0 (IBM), and a two-tailed *P* < 0.05 indicated statistical significance.

## Results

### Patients’ characteristics

Fifty-five patients with primary Meige syndrome who underwent bilateral STN-DBS and finished 5 year’s longitudinal evaluations were included in the current study. They were further divided into response (23 patients) and super-response (32 patients) based on the mean improvement rate of STN-DBS after 5 years. There were no significant differences in sex (*P* = 0.105), age (*t* = 0.816, *P* = 0.386), eTIV (*t* = 0.138, *P* = 0.893), disease durations (*t* = 0.314, *P* = 0.761) and BFMDRS before STN-DBS (*t* = −1.814, *P* = 0.060) between the two groups. The improvement rates of BFMDRS were significantly higher (*t* = −8.598, *P* < 0.001) in the super-response when compared with the response (Table 1).

**Table 1:** Demographics and clinical variables of patients

Groups	Response	Super-response	*P*-value	*T*-value
Subjects	23	32		
Sex (female/male)	19/4	20/12	0.105 ^a^	
Age	57.75 ± 7.16	55.21 ± 10.96	0.386 ^b^	0.816
eTIV	1 411 297.46 ± 142 520.81	1 406 226.38 ± 128 528.68	0.893 ^b^	0.138
Disease duration (year)	5.32 ± 4.69	4.95 ± 4.02	0.761 ^b^	0.314
BFMDRS	18.00 ± 9.35	23.90 ± 13.42	0.060 ^b^	−1.814
BFMDRS improvement rate	0.66 ± 0.14	0.90 ± 0.04	<0.001 ^b^	−8.598

BFMDRS improvement rate was calculated as (BFMDRS before STN-DBS − BFMDRS after STN-DBS)/BFMDRS before STN-DBS. Values are shown as mean ± SD.

BFMDRS, Burke–Fahn–Marsden dystonia rating scale, eTIV, estimated total intracranial volume; STN-DBS, bilateral deep brain stimulation of the subthalamic nucleus.

^a^ *P*-values are calculated using the χ^2^ test.

^b^ *P*-values are calculated using the two-tailed independent sample *t*-test.

### Longitudinal outcomes of BFMDRS

**Figure 2:**
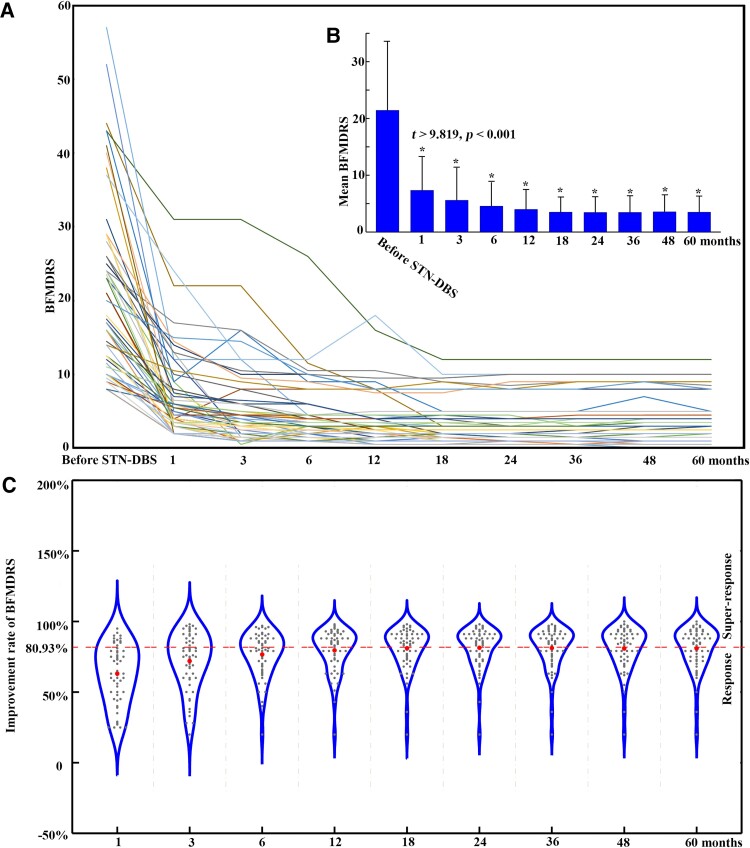
**BFMDRS and improvement rates during 5 years of bilateral STN-DBS in the Meige syndrome.** (**A**) BFMDRS for each patient with Meige syndrome before and after STN-DBS for 5 years (*n* = 55). (**B**) Mean BFMDRS at each follow-up time was compared with mean BFMDRS before STN-DBS. Paired two-sample *t*-tests were performed between mean BFMDRS before STN-DBS and those at each time (1, 3, 6, 12, 18, 24, 36, 48 and 60 months) in 55 patients with Meige syndrome. Asterisk represents *P* < 0.005, Bonferroni corrected for multiple comparisons. (**C**) The mean improvement rates of BFMDRS for patients with Meige syndrome after STN-DBS for 5 years. The improvement rates were calculated as (BFMDRS before STN-DBS − BFMDRS after STN-DBS)/BFMDRS before STN-DBS. The patients with Meige syndrome were further divided into two groups based on their clinical improvement rate after 5 years of STN-DBS, including response (lower than mean improvement rate = 80.93%, dashed line) and super-response (>80.93%).

The BFMDRS for each patient were evaluated at 1, 3, 6, 12, 18, 24, 36, 48 and 60 months after STN-DBS (Fig. 2A). They were significantly decreased at each time when compared with BFMDRS before STN-DBS (*t* > 9.819, *P* < 0.001 for all, Fig. 2B). The improvement rates of BFMDRS were 63%, 71.97%, 76.64%, 79.51%, 81.02%, 81.36%, 81.16%, 80.80%, and 80.93% at 1, 3, 6, 12, 18, 24, 36, 48 and 60 months, respectively, and remained steady across 1–5 years (Fig. 2C).

### Results of VBM

**Figure 3:**
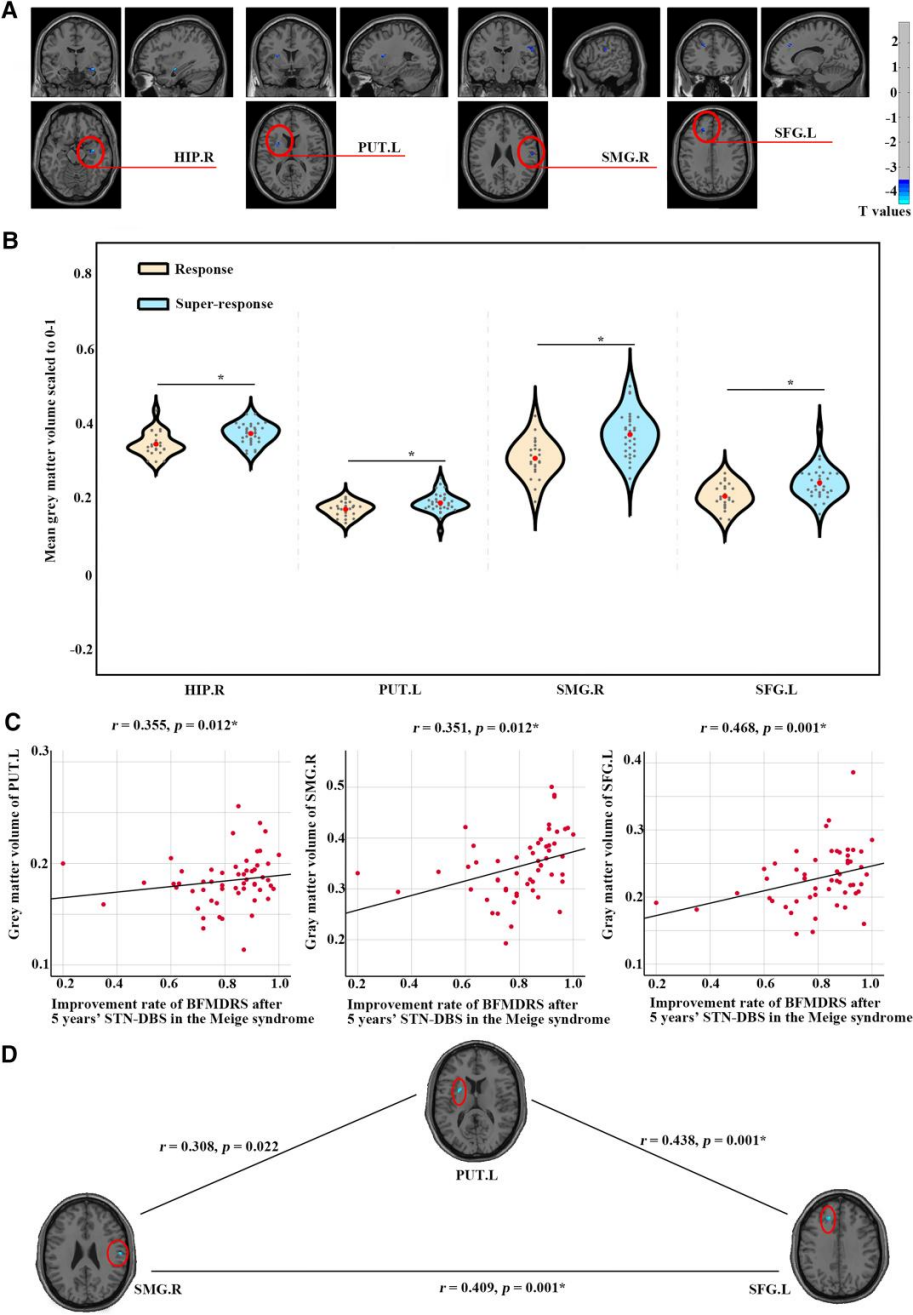
**Difference of GMV between response (*n* = 23) and super-response (*n* = 32).** (**A**) The GMV of HIP.R, PUT.L, SMG.R and SFG.L was decreased in the response when compared with super-response. The results were obtained using two-sample *t*-tests of whole-brain voxel-wise grey-matter maps between response and super-response with age, sex, eTIV, disease duration and BFMDRS as covariates, and thresholded with a voxel-wise *P* < 0.001, cluster size of 40. (**B**) The mean GMV was lower in the response (*n* = 23) when compared with super-response (*n* = 32) using two-sample *t*-tests. Asterisk represents *P* < 0.05/4 = 0.0125, Bonferroni corrected for multiple comparisons. The small dots within the violin plot represent the GMV for each subject, whereas the large dots indicate the mean GMV for each group. (**C**) The GMV was positively correlated with the improvement rate of BFMDRS after STN-DBS for 5 years in the Meige syndrome (*n* = 55) using Pearson’s correlation. Asterisk represents *P* < 0.05/4 = 0.0125, Bonferroni corrected for multiple comparisons. (**D**) Pearson’s correlations were performed between any two regions of GMV in the PUT.L, SMG.R and SFG.L in the Meige syndrome (*n* = 55). Asterisk represents *P* < 0.05/4 = 0.0125, Bonferroni corrected for multiple comparisons.

We identified significantly lower GMV in the right hippocampus (HIP.R, *t* = −4.491, *P* < 0.001), left putamen (PUT.L, *t* = −4.100, *P* < 0.001), SMG.R (*t* = −3.828, *P* < 0.001) and SFG.L (*t* = −4.012, *P* < 0.001) in the response when compared with super-response (Fig. 3A and B, Table 2).

**Table 2:** Brain regions showing significantly decreased GMV in the response when compared with super-response

Brain regions	Abbreviations	Cluster size	MNI coordinates	Peak intensity (*T*-values)	Cohen’s *d*
Right hippocampus	HIP.R	82	(34, −9, −15)	−4.491	0.420
Left putamen	PUT.L	48	(−27, 4, 13)	−4.100	0.350
Right supramarginal gyrus	SMG.R	53	(58, −15, 25)	−3.828	0.305
Left superior frontal gyrus	SFG.L	51	(−15, 34, 34)	−4.012	0.335

### Results of correlations

The improvement rates of BFMDRS after STN-DBS for 5 years were not significantly correlated with age, sex, eTIV and disease durations, but positively correlated with the BFMDRS before STN-DBS (*r* = 0.322, *P* = 0.016) in the Meige syndrome. In addition, improvement rates of BFMDRS after STN-DBS for 5 years were positively correlated with the GMV of PUT.L (*r* = 0.355, *P* = 0.012), SMG.R (*r* = 0.351, *P* = 0.012) and SFG.L (*r* = 0.468, *P* = 0.001) in the Meige syndrome with age, sex, eTIV, disease durations and BFMDRS before surgery as covariates (Fig. 3C). However, the improvement rates of BFMDRS after STN-DBS for 5 years were not positively correlated with the GMV of HIP.R (*r* = 235, *P* = 0.101).

The GMV of PUT.L was significantly correlated with GMV in the SFG.L (*r* = 0.438, *P* = 0.001), the GMV of PUT.L was correlated with GMV in the SMG.R (*r* = 0.308, *P* = 0.022) and the GMV of SMG.R was significantly correlated with GMV in the SFG.L (*r* = 0.409, *P* = 0.001; Fig. 3D), suggesting that they may be involved in the same network.

### Results of ROC and SVM

**Figure 4:**
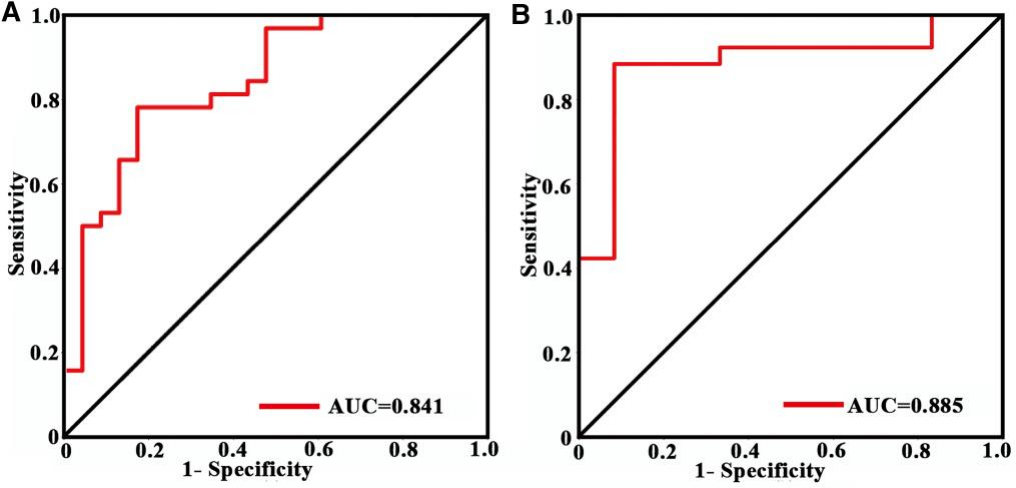
**Prediction analyses.** ROC curves showed good performance for combined features of GMV in the PUT.L, SMG.R and SFG.L in classifying STN-DBS outcomes after 5 years in the Meige syndrome (*n* = 55) using SPSS (**A**) and SVM (**B**).

Regarding predictive performance, the GMV in the PUT.L, SMG.R and SFG.L presented a reliable classification ability in distinguishing response and super-response (ROC: AUC = 0.841 and SVM: AUC = 0.885, Fig. 4).

## Discussion

In the current study, we identified that the longitudinal outcomes of STN-DBS in the primary Meige syndrome were near to 80.93% after 1 year and remained steady across 1–5 years. Based on this rate, all the patients were subdivided into response and super-response. Further VBM analyses revealed significantly lower GMV in the HIP.R, PUT.L, SMG.R and SFG.L in the response when compared with super-response. The GMV in the PUT.L, SMG.R and SFG.L was not only positively correlated with improvement rates of BFMDRS after STN-DBS for 5 years in the primary Meige syndrome, but also presented reliable classification abilities in distinguishing response and super-response.

After STN-DBS stimulations, most patients responded immediately with a mean improvement rate of 63%, reached peak effects after 12 months with a mean improvement rate of 79.51% and remained stable for up to 5 years. These outcomes were in aligns well with previous reports showing improvement rates ranging from 56 to 93%.^6^ At some extent, they are better than those improvement rates in some previous studies, especially one meta-analysis which only showed improvement of 46.4% in BFMDRS motor score at the last follow-up visit based on three centres with 28 patients.^3^ One previous study showed that dystonia movement subscores in 14 consecutive patients improved from 19.3 ± 7.6 before surgery to 5.5 ± 4.5 at final follow-up (28.5 ± 16.5 months) with a mean improvement of 74%.^9^ Another study with a cohort of 30 patients with primary Meige syndrome showed that bilateral STN-DBS led to a 63.0 and 66.8% reduction in the BFMDRS movement scores at 1 and 3 years after neurostimulation, respectively.^8^ To our best knowledge, our study is the longest with nine time points follow-up to 5 years in a relatively larger sample size of 55 patients with primary Meige syndrome who finished bilateral STN-DBS. Collectively, these longitudinal results provided extra evidence to support the fact that bilateral STN-DBS is an effective and safe treatment for primary Meige syndrome.

Based on whole-brain VBM, we identified lower GMV in the HIP.R in the response when compared with super-response. This result was inconsistent with one previous study, which demonstrated significant reductions of GMV in the hippocampus in 27 patients with craniocervical dystonia when compared with 54 healthy controls based on whole-brain VBM.^25^ However, the GMV in the HIP.R was not correlated with improvement rates of BFMDRS in the Meige syndrome. This result might imply that the GMV in the HIP.R might not be associated with motor symptom improvements of Meige syndrome. Besides motor symptoms, Meige syndrome has been commonly exhibited non-motor symptoms, such as depression, anxiety, cognitive impairments and sleep disorders, with a higher incidence than in the general population.^26,27^ As we all know, the hippocampus has been commonly suggested to be involved in processing emotional-related information, such as emotion retrieval, encoding emotional memories and forming episodic representations of the emotional significance.^28-30^ Bilateral hippocampal volume reduction has been reported in patients with depression disorders, and even related to the severity of symptoms, the course of disease and the number of recurrences.^31^ In addition, Meige syndrome with depression symptoms exhibited decreased GMV in the hippocampus when compared with those without depression, suggesting that the hippocampus plays an important role in the pathogenesis of depression in patients with Meige syndrome.^32^ However, whether STN-DBS in the Meige syndrome could release depression symptoms remains controversial. Some studies showed that depression status did not yield significant amelioration in the primary Meige syndrome after STN-DBS,^8^ or even suggested as a stimulation-related adverse effect of STN-DBS in the dystonia based on a systematic review,^33^ whereas many other studies reported an improvement rate of depression.^12,34^ Recently, one study showed that there were larger improvements in the STN-DBS than the GPi-DBS in mean score changes on the 17-item Hamilton depression rating scale in the Meige syndrome.^11^ It is a pity that depression status was not evaluated during programming at each follow-up in the current study. Thus, no relationship was performed between the GMV in the HIP.R and depression release rates in the Meige syndrome. Further longitudinal design with evaluating non-motor symptoms was highly warranted. Anyway, it is possible that the decreased GMV in the HIP.R might be associated with the depression release of Meige syndrome after STN-DBS.

We also identified lower GMV in the PUT.L, SMG.R and SFG.L in the response when compared with super-response, which were positively correlated with improvement rates of BFMDRS in the Meige syndrome. These results suggested that the GMV in these regions might be associated with motor symptom improvements of Meige syndrome after STN-DBS. The putamen is a key region of basal ganglia-cortical circuits,^35^ which has been commonly reported to be pathologically altered in the dystonia. Structural abnormalities, such as decreased GMV,^36,37^ as well as functional abnormalities, such as reduced functional connectivity,^38,39^ reduced nodal degrees^40^ and decreased serotonin receptor binding^26^ in the putamen, have been widely reported in idiopathic dystonia or Meige syndrome. Clinically effective botulinum toxin treatment could reduce the activation of the contralateral putamen during forehead stimulation in idiopathic orofacial dystonia.^41^ Functional connectivity was decreased between the PUT.L and the SMG.R in the blepharospasm/orofacial dystonia.^39^ In addition, there were established connections between the posterior putamen and the SFG.L (frontal eye fields) in human brains,^42^ which was a brain region known to play a role in the initiation of eye movements and potentially involved in reflexive movement inhibition as well.^43^ Moreover, spasms onset was also coupled with activity within the left posterior putamen and SFG.L in patients with blepharospasm using functional MRI.^44^ Given all these evidence, it is reasonable to speculate that the putaminal-cortical circuits of PUT.L, SFG.L and SMG.R might be associated with spasms release in the Meige syndrome.

To date, some clinical measurements, such as on-surgery age,^14^ severity of disease,^3,7^ disease duration^13^ and electrodes position,^15^ as well as some neurophysiological parameters, such as the average firing rates within GPi,^45^ the peak theta oscillatory activity within GPi^46^ and the volume of activated tissue,^21,47^ have been suggested as potential biomarkers to predict the DBS outcomes. Besides that, several indexes based on preoperative MRI have also been endowed with the possibility to predict the efficacy of DBS surgery. One previous study by Gonzalez-Escamilla *et al*.^20^ proposed that the grey-matter thickness of the regions where structural covariance network topology showed abnormality could significantly stratify the GPi-DBS therapeutic effects in generalized/cervical dystonic patients, another study by Raghu *et al*.^21^ found that the diffusion tensor images-based connectivity between the primary motor putamen and the posterior GPi limb significantly correlated with clinical improvement and could predict the GPi-DBS outcomes in cervical dystonia. These two studies were performed for predicting outcomes of GPi-DBS rather than STN-DBS. To our best knowledge, only one previous study was performed for STN-DBS using preoperative MRI, which showed that the mean GMV of Cluster 5 in the left Lobule VIIIb could predict the efficacy of STN-DBS surgery after 1 year in the Meige syndrome.^22^ It yielded AUC = 0.848 for classifying 31 DBS responders and 10 DBS non-responders. However, this study has been performed to focus on cerebellar VBM, ignoring the useful information of the cerebrum. Moreover, the cerebellar VBM is less widely accepted when compared with whole-brain VBM, since it is highly relied on the quality of structural MRI. In addition, the signal in the cerebellum, especially in the posterior part, is usually not so good.^48^ Here, in the current study, we used the whole-brain VBM and identified that the GMV in the PUT.L, SMG.R and SFG.L could be used as biomarkers to distinguish response and super-response of STN-DBS after 5 years. This result extended the previous study by providing extra preoperative structural biomarkers.

Several limitations should be stressed. First, it is a retrospective study, and no healthy controls or case–control groups were included in the current study. Further parallel controlled trials with longitudinal design are highly warranted. Second, the longitudinal evaluations of non-motor symptoms, such as depression, anxiety, cognitive impairments and sleep disorders, have not been performed in the Meige syndrome. It is impossible to explore the relationship between structural changes and non-motor symptoms, particularly weakening the interpretation of decreased GMV of HIP.R. Third, although the sample size of patients who finished STN-DBS after 5 years is the largest so far, it is an exploratory study on the prediction of STN-DBS, which lacks internal and external validation to test its reliability, robustness and generality. Whether the GMV in the PUT.L, SMG.R and SFG.L could be used as stable biomarkers entails further in-depth investigations with larger sample sizes and more independent data sets. Finally, all the patients who underwent STN-DBS showed very good clinical outcomes, with the lowest mean improved rate of BFMDRS being 63%. This might be related to our recommendation for patients. We only include those patients who might be response well to STN-DBS based on the experience of neurologists after considering the preoperative MRI and symptoms, especially for those with obvious infarcts in the GPi. It is highly warranted to include all the data sets that cover non-responders, poor responders and super-responders to make the results (GMV as a biomarker) more robust and powerful.

## Conclusion

These results suggested that STN-DBS is an effective treatment for primary Meige syndrome with acceptable longitudinal outcomes, and preoperative GMV of putaminal-cortical circuits could be used as potential biomarkers to predict longitudinal outcomes.

## Data Availability

The data supporting the findings of this study are available from the corresponding author upon reasonable request. The T_1_-weighted images of all participants were pre-processed using the Data Processing and Analysis of Brain Imaging toolbox (http://rfmri.org/dpabi). The receiver operating characteristic analyses and statistical analyses were performed using SPSS 25.0 (IBM, Armonk, NY, USA). The Support Vector Machine (SVM) was performed within the LIBSVM (https://www.csie.ntu.edu.tw/~cjlin/libsvm/) using Python 3.
